# Cognitive Differences and Influencing Factors of Chinese People’s Old-Age Care Responsibility against the Ageing Background

**DOI:** 10.3390/healthcare9010072

**Published:** 2021-01-14

**Authors:** Yan Wang, Ruilian Zhang, Shengping Peng

**Affiliations:** 1 School of Public Administration, Hohai University, Nanjing 211100, China; wangyan1108@hhu.edu.cn (Y.W.); pengshengping@hhu.edu.cn (S.P.); 2 Sustainable Minerals Institute, The University of Queensland, Brisbane 4072, Australia

**Keywords:** cognition of old-age care responsibility (COACR), ethics of responsibility, family support, influencing factors, ageing

## Abstract

With the rapid increase in the ageing population (60+) in China since 1999, the problem of supporting the aged is facing increasingly severe challenges. Based on the 2072 valid samples from the Chinese General Social Survey (CGSS) of 2017, a non-sequential multinomial logistic regression model was established to analyse the changing trends and micro-influencing factors of Chinese people’s cognition of old-age care responsibility (COACR). The result shows that offspring responsibility still is a common COACR, but this concept has been gradually weakened and been replaced by the responsibility of the government and the aged. Individual characteristics and relationships with relatives in the models all significantly affect people’s COACR. It is obviously unrealistic for China to completely rely on government and society to provide for the aged. The traditional ethical role of inter-generational responsibility in providing for the aged should be brought into play. Reshaping the inter-generational responsibility ethics of old-age care requires the joint efforts of government, society, families, individuals and other responsible subjects to construct a diversified old-age care service system.

## 1. Introduction

According to the forecast report issued by the Population Division of the United Nations Department of Economic and Social Affairs (2017), the world population will increase from 7.6 billion at present to 9.8 billion by 2050 [1]. With the emergence of low fertility rates, the global ageing problem is becoming increasingly serious. It is estimated in the report that the population aged 60 and over will grow from 962 million at present to 3.1 billion by 2050, and as a proportion of the global population, this population will increase in size from 12.85% to 22%. Among this population, 65% of the elderly aged 60 and over are in Asia [2]. The report notes that the impact of this demographic change will be far-reaching. In the economic sphere, population ageing will have an impact on economic growth, savings, consumption, the labour market, pensions and intergenerational transfer; in the social sphere, population ageing will affect family composition and living arrangements, housing demand, epidemiological problems and the demand for health care services. And in the political sphere, population ageing may affect voting patterns and political representation. As the most populous country in the world, China’s ageing problem is particularly serious. Since China became an ageing society in 1999, the ageing population has been rising steadily [3]. According to the authoritative data released by the National Bureau of Statistics in January 2018, by the end of 2017, the population aged 60 and over in China had reached 240.9 million, accounting for 17.3% of the country’s total population [4]. Among this population, 158.31 million were aged 65 or above, accounting for 11.4% of the total population. Population ageing will become increasingly normal, which may have more negative effects on labour productivity [1,2]. Although China started to fully implement the two-child policy [1] in 2016, the adjustment of its birth policy cannot fundamentally alleviate the gap of the old-age pension [2]. China’s problem of supporting the aged is facing increasingly severe challenges, and “who should provide for the aged” has become a problem that people must consider. 

Under the influence of the welfare pluralism that emerged in Western countries in the 1970s, China has built a pluralistic old-age security system coordinated by government, family and society. How multiple old-age care policies can be targeted at specific groups and the extent to which they can they be implemented effectively depend on residents’ cognition of old-age care responsibility (COACR). The introduction of market mechanisms in social reform has led to the differentiation of social groups. How does the old-age security system prevent the waste of old-age care resources and enable the groups in greatest need of help to receive this help? This requires residents’ COACR to be clear. Consequently, three aspects will be explored in this paper: the first is residents’ COACR; second, the factors affecting residents’ COACR; third, corresponding countermeasures and suggestions proposed on the basis of the first two aspects.

## 2. Literature Review

It has been exactly 20 years since China became an ageing society, and the country has achieved many fruitful results. Academic research mainly addresses the following three aspects.

The first is the change in the traditional norm of old-age care and its influencing factors. For thousands of years, China has followed the traditional norm of “raising offspring for old age”. However, Cui, Xu and Wang found in a survey of 209 elderly people living in homes and nursing homes in Shanghai that dissenters accounted for 43.6% of the elderly people surveyed, which fundamentally conflicts with the deep-rooted norm of “raising offspring for old age” [3]. Ge, on the basis of the low fertility rate in Shanghai, believed that the norm of “raising offspring for old age” has been basically eliminated [4]. Zhu and Ouyang also believed that the perception of traditional old-age care has undergone fundamental changes, the concept of independent old-age care has gradually formed, and more attention has been paid to spiritual old-age care [5]. Many scholars have studied changes in old-age care concepts among specific populations, such as families with an only child [6], those that lost an only child [7], migrant populations [8], land-expropriated farmers [9], urban and rural residents [10], and the young and old [11].

With regard to factors influencing the norm of “raising offspring for old age”, Cui, et al. believed that gender, age, educational level, marital status and health status did not affect this norm [3]. In a survey of over 10 provinces and more than 1000 farmers in China, Yu also found that neither sex nor marital status affect the norm. However, the author found that with regard to farmers’ individual characteristics (age, nation, health status, educational level), family characteristics (family relationship, whether there are sons, family size), community characteristics (occupational type) and district characteristics (village geographical environment), nine factors have a significant impact on farmers’ norm of “raising offspring for old age” [12]. Based on an analysis of the tracking survey data of China’s elderly society in 2014, Wang found that elderly people in rural areas and those with middle school education and above, self-rated good health, large family size, better housing and greater income were more likely to agree with the norm of “raising offspring for old age” [13].

The second issue is the willingness to choose a mode of old-age care and its influencing factors. Currently, there are still differences in the classification of old-age care modes and classification standards. Based on previous studies, old-age care modes can be summarised into two main types. The first is mainly divided into self-support, family support and social support modes according to the subject of old-age care responsibility (OACR). The second type is mainly divided into home-based endowment (or community home endowment) and organisation endowment according to living place.

Considerable research has been conducted in China on this topic. According to the first category, the combination of the self-support and family support modes is the most popular in rural areas [14]. In underdeveloped areas, self-support is the transitional mode of rural old-age care, and social support is the final mode of rural old-age care [15]. For the elderly in the city, relying on their offspring is still the first choice for the elderly, who prefer to live with their children rather than living in old-age care institutions [16]. However, some scholars hold the opposite view, suggesting that most urban residents are less dependent on their offspring for old-age care and that reliance on self-support and social support may be the mainstream form of old-age care in the future [17]. According to the second category, the vast majority of rural elderly people are not willing to choose an organization endowment [18]. The analysis shows that traditional attitudes towards residence in nursing homes and different lifestyle between residing at home and in nursing homes, explain their reluctance to choose nursing homes [19]. Male residents are more likely than female residents to expect home-based endowment [17,20]. Parents of only offspring are likely to choose home-based endowments when they are healthy and to choose organization endowments when their health is poor [20]. The elderly people in underdeveloped areas tend to choose the mode of home-based communal medical care combined with old-age services [21]. However, an increasing number of elderly people have gradually come to accept organization endowment [22].

In terms of influencing factors, according to the first category, family structure and inter-generational exchange significantly influence the choice of the old-age care mode for the elderly; that is, the closer the relationship between the elderly and their offspring and the more elderly pay for their offspring, the more inclined they are towards family support [23,24]. The better the economic status and social security of the elderly are, the lower their family support intention will be, and the higher their social support intention. Moreover, the influence of economic status and social security on the willingness of urban residents to provide for the aged is more significant than that of rural residents [25]. Compared with older unmarried men in rural areas, married men are more inclined towards family support [26], whereas unmarried men are more inclined towards self-support and reliance on old-age insurance [27]. According to the second classification, government behaviour (pension system operation, institutional service supervision) has a significant positive impact on residents’ organization endowment intention [28], whereas social support of the elderly (acquaintance scale, party time, popularity self-identity, loneliness connotation, etc.) is negatively correlated with residents’ organization endowment intention [22]. In addition, the old-age care mode of the elderly is affected by their marital status, income level and health status [22]. The more harmonious the inter-generational relationship of the elderly is, the lower their organization endowment intention will be; conversely, the higher the socioeconomic status of the elderly is, the higher their organization endowment intention will be [29]. It is not clear on the subject of old-age care responsibility (OACR) in the existing literature. There are many members in the family, but it is unclear who is responsible for the old-age care. The subject of OACR on the social support is less clear. Moreover, the influencing factors research, such as social interaction are not sufficient in the existing literature. Willingness to choose old-age care mode and its influencing factors is shown in Table 1.

The third issue involves the relationship between studies of different old-age care models. With the gradual evolution of Chinese families from having an economic function to having an emotional function, the traditional old-age care mode that relies solely on offspring is increasingly unable to meet the needs of the elderly [30]. Research in China shows that the vigorous implementation of the new rural insurance [2] has a far greater stimulating effect on the supply of old-age pension resources for the elderly than the restraining effect of the reduction in current disposable income produced by insurance [31]. Social support therefore has a certain degree of substitution for traditional family support, but it has not fundamentally changed the core status of Chinese family support [32]. Although social support improves the economic independence of the elderly in terms of financial support, it weakens the emotional communication between offspring and their parents in terms of spiritual comfort and reduces the number of times that offspring visit their parents. This effect is greater in families with sons than in families with daughters [33]. Feng and Gao (2013) suggest that the three typical rural old-age care modes, new rural insurance, family support and social support, have different degrees of integration [34].

At present, there is little research on cognition of old-age care responsibility (COACR) in China. Focuses on the COACR of rural elderly people in China and believes that rural elderly people with different characteristics have different cognition and that pension insurance has a limited impact on their cognition [35]. After surveying eight counties and cities in Hunan province, Luo found that land-expropriated farmers believed that local governments, families and village collectives should bear all or part of the responsibility for old-age care, accounting for 73.8%, 69.6% and 44.3%, respectively [9]. These studies combine theory and empirical evidence and offer important reference values to future studies, but there are also some deficiencies. First, the research focus is too narrow, and the research content is not sufficient. Second, the research focuses on a particular group or a certain local area, such as families that lost an only child, only-child families, land-expropriated farmers, rural residents, and migrant populations, whereas there are few studies on the overall COACR in China. Third, existing studies focus on cross-sectional data analysis rather than tracking the changes and trends of old-age care concepts. By examining the whole of China, not only based on the 2017 Chinese General Social Survey (CGSS) data but also with reference to the data from 2010 and 2015, this study can grasp the transformation of Chinese residents’ concept of old-age care and its influencing factors as a whole and can provide an important reference for the Chinese government to formulate scientific and reasonable old-age care policies.

## 3. Research Methods

### 3.1. Data Source

The data used in this paper are from the 2017 CGSS sample questionnaire. The survey adopted a multistage and stratified sampling method. First, 105 primary sampling units (100 counties (districts) and five large cities) were selected nationwide. Second, village/neighbourhood committees (four for each county (district) and 80 for each large city) were selected from each selected primary unit. Then, families were selected from each village/neighbourhood committee. Finally, one person was randomly selected from the selected families for an interview, and a total of 12,582 survey samples were obtained. In this paper, 2072 samples were ultimately selected after removing the samples with missing values.

### 3.2. Values of Variables

All variables in this paper are divided into dependent variables and independent variables, as shown in Table 2. The determination of dependent variables and categorization of the independent variables are selected based on the following two factors. One consists of the relevant references; the other is the traditional Chinese old-age care culture background and actual national conditions.

The dependent variable is residents’ COACR. To measure the questionnaire item “Who do you think should be responsible for the old-age care of the elderly with offspring?”, there are four alternatives, “mainly responsible by government”, “mainly responsible by offspring”, “mainly responsible by the elderly themselves” and “the government/offspring/the aged(the Three Parties) sharing responsibility respectively”.

The independent variables are the influencing factors of residents’ COACR, which are mainly divided into the following five categories. Because the number of people in some items of the independent variables in the questionnaire is too small, in order to make full use of the data, many items are merged. The premise is that the measurement category of variables is not changed, and the statistical results are not affected.

First, the birth year variable is selected to measure the characteristic of age. Because the centralization of power was obvious after independence in 1949, the social movement initiated by central policy had more influence on society than any other factor [36]. Therefore, the birth age of the respondents is divided into pre–1948, 1949–1965, 1966–1978 and after 1979, based on major social events.

Second, the Hukou [3] type variable is selected according to geographical characteristics. It is merged into rural Hukou and urban Hukou.

Third, the variable of self-evaluation of family economic status is selected according to economic characteristics. Five responses ranging from “far below the average” to “far above the average” are merged into 3 items: “low”, “medium” and “high”.

Fourth, the educational level variable is selected according to educational characteristics. Thirteen ratings ranging from “no education” to “graduate student or above” are merged into 5 ratings ranging from “no education” to “higher education”.

Fifth, the variable of contacts with relatives is selected according to the social interaction characteristic. Eight items from “daily contact” to “never contact” are merged into 2 items: “not close” and “close”.

### 3.3. Sample Description

The 2072 respondents, 45.5% were male and 82% were married as shown in Table 3. Those born before 1948, 1949–1965, 1966–1978 and after 1979 accounted for 21%, 51.5%, 23.6% and 3.9% respectively. The rural residents accounted for 57.5%. Self-rated low, medium and high family economic status accounted for 46.8%, 46.7% and 6.5% respectively. The uneducated, primary school educated, middle school educated, high school educated and tertiary education accounted for 15.2%, 28.2%, 30.2%, 17.3% and 9.1% respectively. People who were close to relatives (PWCR) accounted for 96.1%. For the question “Who do you think should be responsible for the old-age care of the elderly with offspring?”, the answers were “mainly responsible by government”, “mainly responsible by offspring”, “mainly responsible by the elderly themselves” and “the three parties sharing responsibility”, which were 14.6%, 47.1%, 7.7% and 30.6% respectively.

### 3.4. Model Selection

On the basis of understanding the overall changes of COACR in China, this paper uses the CGSS 2017 data and a logistic regression model to study the micro factors affecting COACR. In this paper, there is no sequential relationship among the four values of dependent variables, so the non-sequential multinomial logistic regression model is applicable. The basic idea of this model is to take a certain value of dependent variable as the reference group (the maximum default value of SPSS is the reference group), and other values can be compared with it to fit (K-1) generalized logistic regression models (K being the number of values of dependent variables). When there are two values of the dependent variable, the non-sequential multinomial logistic regression is equivalent to the binary logistic regression. Therefore, the non-sequential multinomial logistic regression can be regarded as an extension of the binary logistic regression. Taking Y = 4 as the reference group, three models are fitted with M independent variables as follows.

(1) $$\mathrm{logit}P_{1}=\ln\left(\frac{P_{1}}{P_{4}}\right)=\alpha_{1}+\beta_{11}x_{1}+\beta_{12}x_{2}+\cdots +\beta_{1m}x_{m}$$

(2) $$\mathrm{logit}P_{2}=\ln\left(\frac{P_{2}}{P_{4}}\right)=\alpha_{2}+\beta_{21}x_{1}+\beta_{22}x_{2}+\cdots +\beta_{2m}x_{m}$$

(3) $$\mathrm{logit}P_{3}=\ln\left(\frac{P_{3}}{P_{4}}\right)=\alpha_{3}+\beta_{31}x_{1}+\beta_{32}x_{2}+\cdots +\beta_{3m}x_{m}$$

In the above formulas, *P*_4_ is the occurrence probability of the reference group, *P*_1_, *P*_2_ and *P*_3_ are the occurrence probabilities of other groups, and there should be *P*_1_ + *P*_2_ + *P*_2_ + *P*_4_ = 1. α is the intercept; β is the slope. The odds ratio (OR) is often used to explain various regression coefficients in regression model analysis. “EXP (B)” is the OR value of the corresponding variables. In this paper, SPSS 25.0 software is used to conduct an in-depth analysis of COACR of different social groups using non-sequential multinomial logistic regression model.

**Figure 1:**
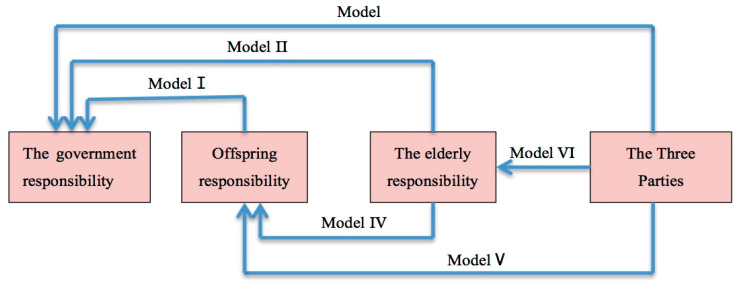
Reference Groups of Six Models. Source: designed by the authors.

Because there are four values of dependent variables in this paper, a total of 12 models need to be fitted if each two variables are compared. However, because there is an opposite-sign relationship between the coefficients of each two variables, only half of the models need to be listed. Moreover, the coefficients of the other six models can be deduced from the coefficients of the six models listed. If the coefficient is known, the OR value will be known. Government responsibility, offspring responsibility and responsibility by the elderly are taken as the reference groups, and finally, six multinomial regression models are selected (Figure 1). Among them, taking government responsibility as the reference group, there are four values of dependent variables, and models Ⅰ, Ⅱ and Ⅲ can be fitted. Taking offspring responsibility as the reference group, there are three values of dependent variables, and models Ⅳ and Ⅴ can be fitted. Taking the elderly responsibility as the reference group, there are two values of dependent variables, and model Ⅵ can be fitted.

## 4. Results and Discussion

### 4.1. Overall Situation

**Figure 2:**
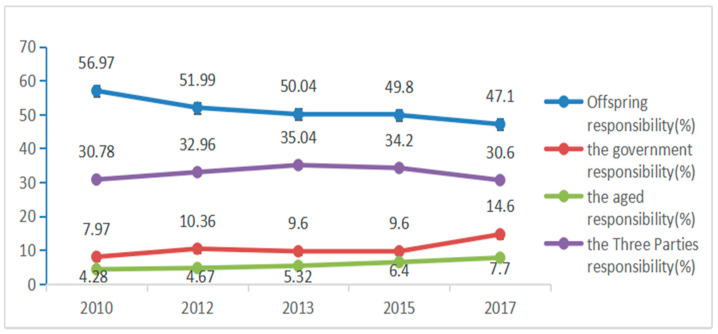
Changes in COACR in China during 2010–2017. Data source: [37] and the authors’ statistics.

Based on the existing literature [37] and the sample description of COACR in 2017 in Section 3.3, the frequency change of COACR in China during 2010–2017 can be obtained (Figure 2). The statistical result shows that residents have significant differences in COACR. The majority of people in the sample believe that offspring should be responsible for old-age care, indicating that Chinese residents have a unique preference for offspring to be responsible for old-age care. Second, the Three Parties are responsible. The aged account for the smallest proportion, which is probably related to China’s long-standing concept of “raising offspring for old age”. However, the authors also find that with the passage of time, the improvement of social and economic conditions and the gradual improvement of the social security system, people’s idea of old-age care are changing. Although most people still believe that offspring should be responsible for providing for the aged, this trend is decreasing year by year. The concept of “The Three Parties share equally” rises up first and then falls down. Meanwhile, the believe that the government and the aged should be responsible is increasing year by year. In particular, the rise of the “government responsibility” is most obvious. This trend indicates that people have begun to gradually recognize other ways of old-age care modes besides offspring responsibility.

### 4.2. Results of a Multinomial Logistic Regression Analysis of COACR 

Table 4 shows that all independent variables have different degrees of influence on the respondents’ COACR. First, in terms of birth year, in model I and model III, those born before 1948 attribute OACR to offspring and the Three Parties 0.23 times (e^−1.478^) and 0.3 times (e^−1.195^) respectively as much as those born after 1979. It indicates that those born before1948 prefers government to assume OACR. Those born in 1949–1965 attribute OACR to offspring and the Three parties 0.3 times (e^−1.201^) and 0.4 times (e^−0.928^) as much, respectively, as those born after 1979. This indicates that those born in 1949–1965 prefer the government to assume OACR. Obviously, all those born before 1965 have the same orientation in COACR, that is to say, they think that the OACR mainly rests with the government.

In model IV, those born before 1948 and in 1949–1965 attribute OACR to the aged 8.26 times (e^2.111^) and 3.97 times (e^1.38^) as much, respectively, as those born after 1979. It indicates that those born before 1965 prefers the aged to assume OACR themselves.

Second, in terms of Hukou type, in model I and III, rural residents think that the probabilities of attributing OACR to offspring and the Three Parties are 4.58 times (e^1.522^) and 1.72 times (e^0.542^) that of urban residents, respectively. It indicates that rural residents prefer offspring or the three parties to bear OACR.

In model IV and model V, rural residents think that the probabilities of attributing OACR to the elderly and the Three Parties are 0.2 times (e^−1.606^) and 0.38 times (e^−0.98^) that of urban residents, respectively. It indicates that rural residents prefers offspring to bear OACR.

In model VI, rural residents think that the probabilities of attributing OACR to the Three Parties 1.87 times (e^0.626^) that of urban residents, respectively. This indicates that rural residents prefer the Three Parties to bear OACR.

Third, in terms of self-evaluation of family economic status, in model II, people with low self-rated family economic status think that the probabilities of attributing OACR to the elderly 0.51 times (e^−0.672^) that of people with high self-rated family economic status, respectively, which indicates that people with low self-rated family economic status prefer government to bear OACR.

Fourth, in terms of educational level, in model I, the uneducated attribute OACR to offspring 1.96 times (e^0.672^) as much as those who are highly educated, respectively. It indicates that that the uneducated prefers offspring to bear OACR.

In model V, the people of primary school education level attribute OACR to the Three Parties 0.62 times (e^−0.479^) as much as those who are highly educated, respectively. It indicates that people of primary school education level prefer offspring to bear OACR.

In model VI, people of middle school education level attribute OACR to the Three Parties 0.81 times (e^−0.213^) as much as those who are highly educated, respectively. It indicates that people of middle school education level prefer the elderly themselves to bear OACR.

Fifth, in terms of contacts with relatives, in models II, PWCR adopt attributions of OACR to the elderly at levels 1.63 times (e^0.489^) greater, respectively, than people who are not close to relatives. It indicates that PWCR prefer the elderly to support themselves.

### 4.3. Analysis of the Influencing Factors of COACR

The above analysis shows that residents’ COACR tends to be diversified. In model I, mid-older adults and people with low self-rated family economic status tend to think that the government should bear OACR rather than their offspring. (1) Today, people born before 1965 are at least 55 years old, and 73.9% of them have less than a middle school education level. Under the planned economic system at that time, they began to work around the age of 16. From the founding of New China to workers’ lay-offs and unemployment from state-owned enterprises in the mid- and late 1990s, they contributed their youth and made important contributions to the industrialization process of the country but did not accumulate much wealth for future generations. As Guo said, this generation of old people is in a very difficult situation. When these individuals are in their primes of their lives, they are deprived of their wealth. It is impossible to accumulate family wealth for their offspring. In the era in which the pursuit of prosperity became possible, they were already dying and old. They have no family business, property or honour to pass on to the next generation. In the inter-generational exchange, they give nothing in return, which means that the economic basis of the pay-return inter-generational ethical relationship has changed [38]. (2) People with low self-rated family economic status have no choice but to regard the government as the main party responsible for OACR because there is no other object upon which to rely. They also do not rely on other social connections. The cross-linked table analysis of age, self-evaluation of family economic status and Hukou shows that 16.6% of urban residents and 29.8% of rural residents born before 1965 belong to people with low self-rated family economic status. In the process of economic reform, they have fallen from a stratum with senior middle social status to a vulnerable group in society and become “the group with relatively damaged interests” in reform. They are the generation that bears the risks and costs of social transformation. In this sense, society owes them. Although the traditional inter-generational model follows the “return” or “feedback” model, in the planned economy era in which the trajectory of individual life is significantly affected by the state, the relationship between the individual and the state also follows the equitable logic of paying and rewarding. Currently, this group is facing the dilemma of providing for the aged. It is a rational cognitive choice based on fair exchange to require government to assume the main OACR.

In model I, rural residents and the uneducated tend to think that offspring should bear OACR rather than the government. Family support has always been a tradition in China; it is still the dominant mode among the many current old-age care modes. (1) The cross-linked table of Hukou and OACR shows that 58.1% of rural residents uphold the concept of supporting the aged by offspring. This finding indicates that the concept of family support still occupies a dominant position in rural areas. Some scholars have found that the one-child population policy and the out-migration of agrarian individuals have dramatically reduced the traditional rural population and even created “hollow villages”. They suggest that rural filial piety has declined or even collapsed, leading family old-age care functions to decline. However, the results of this paper suggest that traditional family support is still common in most rural areas; otherwise, the vast majority of farmers would not still expect their offspring to provide for them. He’s research notes that relatives play the most important role in providing support for the elderly in rural areas. Among relatives, offspring play the most important role. Some of the major practical and emotional support comes from offspring, especially from sons [39]. (2) It can be seen from models I, V and VI in Table 3 that the data results change significantly from primary school education to middle school education. It is obvious that the attitude of the middle school educated towards OACR shifts from offspring support to self-support. The middle school educated group comprises the largest proportion of population (30.2%) among the education groups in this paper; it accounts for the largest proportion of all migrant workers [4] in China and is most affected by the outside world. According to the China Industry Information Network, 59.2% and 58.6% of migrant workers in China had a middle school education in 2016 and 2017 respectively. Compared with the less educated, the middle school educated live longer in cities and are more susceptible to the influence of urban residents’ gradual tendency towards the self-support mode. Compared with the more educated, they have lower economic status and income, and they cannot provide more old-age security for the elderly.

**Figure 3:**
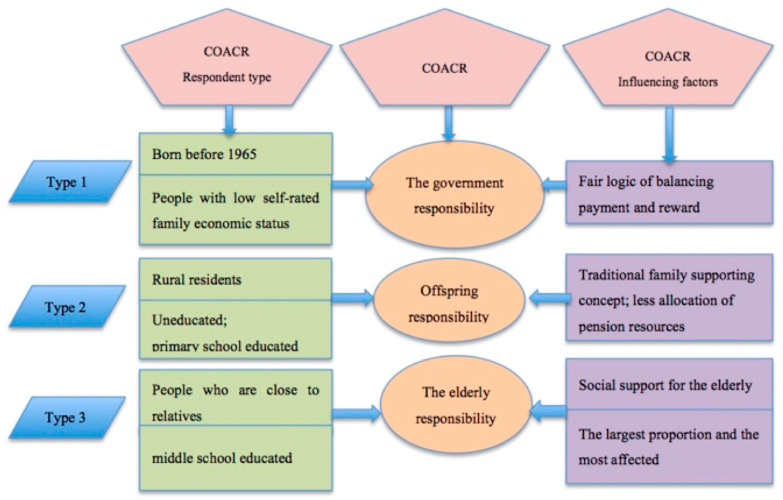
Differences of COACR and Influencing Factors. Source: designed by the authors. Note: COACR is an abbreviation of cognition of old-age care responsibility.

In model II, compared with those who are not close to relatives, PWCR are more likely to think that the elderly should support themselves. In fact, the elderly in real life are not more frequently burdens on society. Jeanne and Zhang’s survey of the elderly in Shanghai shows that the elderly made significant contributions to the care of the elderly through mutual help and spousal care [40]. It shows that the condition of aged care for the empty nest elderly is relatively good, can take care of themselves and don’t feel lonely in their life [41]. The results of rural data analysis show that the elderly can realize self-support if their relatives can provide social support for them beyond the government. Granovetter noted that a weak linkage, as an important interval bridge in the process of information transmission, is an important way to obtain network resources [42]. According to Granovetter analysis, a “weak relationship” is more powerful than a “strong relationship” in social networks. However, in China, the support given to the elderly in reality is often a “strong relationship” rather than a “weak relationship”. Liu and Ji (2013) suggested that the social network of the elderly is dominated by kinship relationships centred on the spouse and offspring and gradually transfers to a geographical relationship with neighbours and friends [43]. Therefore, more intimate topics and reciprocal relationships occur for the elderly through “strong relationship”, which is where they have gained more social support. In the process of industrialization and urbanization, the traditional extended family has been replaced by the present nuclear family as the main family structure. However, in real life, the mutual support from relatives has not been interrupted due to the reduction in family size. Some scholars’ research also proves that the current family is “separated without leaving”. Wang stated in his research on the changes in family types that the old-age care function of individual families has been gradually weakened. The responsibilities and obligations previously assumed by individual family members in the past must be extended to network families and even kinship-circle families [44]. At present, relatives and friends play the role of quasi-family members in social support for the elderly to a certain extent. Fei said that in the local society, regardless of kinship or geographical relationship, there is a circle centred on “oneself”, which is the mutual aid organization in life [45]. Although the current concept of family has faded, the social support provided by relatives has not weakened (Figure 3).

### 4.4. Discussion

At present, there are structural differences in residents’ attitudes towards COACR; that is different groups hold different attitudes towards COACR. First, Hukou in China is more of a difference between urban and rural identity. Compared with urban residents, rural residents are more inclined to assign OACR to their offspring. Goode, one of the representatives of the classical family modernization theory believes that in the process of industrialization, the traditional extended family is gradually transforming into a couple-style nuclear family. This nuclear family is gradually becoming the dominant mode of society, whereas the possibility of the elderly living in the traditional united family is diminishing [46]. Consequently, they have fewer available resources for inter-generational support. This shift will ultimately have a negative impact on the quality of life of rural elderly people who lack the support of fixed-income security and community service. As a public resource, old-age care resources provided by the government are currently more invested in cities. The actual distribution of old-age care resources between urban and rural areas is consistent with the COACR of urban and rural residents. Rural development generally lags behind urban development in social and cultural transformation, and the traditional norm of “raising offspring for old age” is stronger in rural areas than in urban areas. However, some scholars’ field investigations in rural areas show that filial piety and the traditional norm of old-age care is declining in rural areas, and the living conditions of rural elderly people are not encouraging. The data analysis results in this paper show that 36.7% of the people who believe that the government should bear OACR are rural residents. Over the long term, the government should gradually tilt the old-age care resources to rural areas and provide support for the rural elderly. At the same time, the government should provide corresponding policies and support for offspring to assume OACR, so that offspring can gradually eliminate the passive situation of “having the heart but lacking ability” and truly implement OACR. The study finds that immigration reduces the possibility of offspring living with older parents [47]. The results show that adult children’s migration weakens family support for the rural elderly from the aspects of daily care and spiritual comfort which are restricted by space [48]. Both rural residents and the uneducated and the primary school educated emphasis offspring’s OACR, but this norm gradually weakens with the improvement of educational level. This concept also indicates that, with the comprehensive development of social economy and the overall improvement of educational attainment, people will begin to gradually accept other old-age care modes other than offspring’s OACR, which provides a new idea around which China can vigorously develop social support.

Second, mid-older adults over 55 (born before 1965) and people with low self-rated family economic status regard the government as the main source of help for the aged. The life track of the former is closely related to the national policy orientation, and their thinking and ideas still have “institutional traces” of the planned economy era. The latter, after the introduction of the market economy mechanism in the 1990s, had obvious differentiation among social groups and tended to be stereotyped among social strata and gradually fell into the vortex of poverty. It was found that 22% of urban residents and 17.3% of rural residents belonged to both groups in the study. This result coincides with Sun’s (2004) view that the age of laid-off and unemployed workers in the middle and late 1990s tended to be greater than 35 or 40 years old [49]. Inkeles, one of the representatives of the school of social psychology, believes that the existence of “traditional people” who adhere to tradition and do not have the spirit of innovation in developing countries has become an obstacle to the reforms of the national system and social change. [50]. As Cowgill pointed out, modernization tends to weaken the status of the elderly, co-occurring with the decline in the importance of land as a status resource, the extended family diminish and increased mobility in different regions. Moreover, rapidly changing social structures and cultural values will contribute to the decline in the status of the elderly [51]. If declining economic and social status is not changed across the generations, the original relative poverty may change to absolute poverty. It can be imagined that the less able they are to escape poverty, the more dependent they become on the government. Although China’s minimum living security system and old-age insurance system have been greatly improved, efforts should be made to improve the old-age security system, expand the coverage of old-age insurance, increase the participation rate of urban and rural residents, increase investment in these vulnerable groups, and ensure that the elderly can enjoy their old age.

Finally, PWCR and those of middle school education level tend to think that the elderly should provide for themselves during old age. PWCR tend to self-support. This result confirms once again that only when the relationship between relatives is “strong” in China can social support resources be obtained. The role of the network of relatives in supporting the elderly provides a new channel for the exploration of self-support resources. At the same time, the application of social network theory to the practice of self-support for the elderly provides a new idea. Modernization theory holds that the development of social welfare and social security policies has weakened people’s dependence on kinship networks to some extent [46]. However, social welfare may also have a positive impact on inter-generational relations. The “squeeze in” hypothesis put forward by the World Bank in 1994 holds that the supply of national social security policies enables the elderly to have more resources on the basis of guaranteeing their minimum living needs and to provide various forms of assistance and support with their children [52]. On the one hand, the attitude held by middle school educated people that endorses the sharing of responsibility for old-age care has been transferred from offspring’s old-age care to independent old-age care. This finding indicates that the structure of old-age care in Mainland China has undergone profound changes. On the other hand, with the acceleration of urbanization, the solid ice of the urban-rural dual system is gradually melting, and urban-rural integration is tending to occur, which provides important awareness about the need for timely adjustment of old-age care policies and the realization of self-support. The results of Inkeles’ survey in six developing countries show that education is one of the three major factors contributing to personal modernization. School is an important place for individual modernization, and educational level is directly related to modernity. Educational reform is an important part of the country’s modernization. Therefore, it is necessary for the state to increase investment in education and ensure the relative fairness of educational opportunities [50]. The several findings are of great practical significance for exploring self-support strategies and avoiding old-age care risks.

## 5. Conclusions

In the context of ageing, China’s old-age care tasks and circumstances will become increasingly severe, and who will provide for the aged has become a practical problem. Based on CGSS data from 2017, 2072 valid samples were selected, and data from 2010–2015 were tracked to conduct a comprehensive analysis of Chinese residents’ COACR in this paper. This study found that the residents tend to be diversified with regard to COACR. It is still a common element of COACR that offspring provide for the aged, and family support is still the dominant position of COACR in China. However, this cognition of responsibility is gradually weakening and is being replaced by the governmental responsibility, self-responsibility by the elderly and three-party responsibility, and people are increasingly beginning to agree that government and the elderly responsible for the elderly.

Based on China’s current ageing population of 240.9 million and the imperfect old-age security system, it is obviously unrealistic to rely entirely on the government and society for old-age care. As an important part of traditional inter-generational responsibility ethics, offspring’s care for the aged should play an important role. Influenced by traditional Confucianism, elderly Chinese people prefer to be supported by their offspring. Moreover, studies have shown that family support has a stronger marginal effect on health than social support [53]. However, with the progress of the times and the development of the economy, people’s COACR are also gradually changing and tending towards social support. Therefore, it is necessary for the government, society, families and individuals to make joint efforts to rebuild the inter-generational responsibility ethics of old-age care and to build a diversified old-age care service system. On the one hand, we should carry forward the traditional virtue of “you raise me young, I raise you old”, let family members realize the value of the elderly and strive to improve the level of family support to ensure that the elderly with offspring have a good old age. On the other hand, the government should vigorously develop social support and constantly improve various old-age insurance systems; foster the responsibility ethics of offspring to support their parents; provide policy and guidance support; and make social and self-support old-age care a strong backing for the elderly. While improving their economic independence, the elderly can continue to maintain emotional exchanges and interpersonal relationships with their offspring and society.

## Figures and Tables

**Table 1:** Willingness to Choose Old-Age Care Mode and Its Influencing Factors.

No.	Classification Standard	Old-Age Care Mode	Old-Age Care Areas	Choice Willingness	Data Source	Influencing Factors	Data Source
1	The subject of OACR	Self-support, family support and social support	Rural	The combination mode of self-support and family support is the most popular	[14,15]	Family structure and inter-generational exchange; Economic status and social security; Marital status	[23,24,25,26,27]
			Urban	Relying on offspring for old-age care is still favoured, but self-support and social support may be the future direction	[16,17]		
2	The living place	Home-based endowment (or community home endowment) and organization endowment	The whole country	The vast majority of people choose home-based endowment, but organization endowment has gradually been recognized	[17,18,19,20,21,22]	Government action; Social support for the elderly; Inter-generational exchange; Economic status; Marital status; Health status	[22,28,29]

**Table 2:** Variable Assignment.

Variable Category	Variable Name	Variable Assignment
Dependent variable	COACR	The government responsibility = 1, offspring responsibility = 2, The elderly responsibility = 3, The Three Parties’ responsibility = 4
Independent variables	Birth year	Pre–1948 = 1, 1949–1965 = 2, 1966–1978 = 3, after 1979 = 4
	Hukou type	Rural Hukou = 0, urban Hukou = 1
	Self-evaluation of family economic status	Low = 1, medium = 2, high = 3
	Educational level	Uneducated = 1, primary school education = 2, middle school education = 3, high school education = 4, tertiary education = 5
	Contacts with relatives	Close = 0, not close = 1

**Table 3:** The Sample description.

Variable	Percent (%)
Male	45.50%
Married	82%
Before1948, 1949–1965, 1966–1978, after 1979	21%, 51.5%, 23.6%, 3.9%
Self-rated low/medium/high family economic status	46.8%, 46.7%, 6.5%
Uneducated, primary/middle/high school educated	15.2%, 28.2%, 30.2%, 17.3%, 9.1%
Close to relatives	96.10%
Mainly responsible by government/offspring/the elderly themselves/the three parties	14.6%, 47.1%, 7.7%, 30.6%
Total sample	2072

**Table 4:** Results of Multinomial Logistic Regression Analysis of COACR.

Category	Variable Name	Model I	Model II	Model III	Model IV	Model V	Model VI
		(Reference Group: The Government Responsibility)			(Reference Group: Offspring Responsibility)		(Reference Group: The Elderly Responsibility)
		Offspring	The Elderly	The Three Parties	The Elderly	The Three Parties	The Three Parties
Age characteristics	Birth year (after 1979)						
	Pre–1948	−1.478 ***	0.633	−1.195 **	2.111 ***	0.284	−1.828 *
	1949–1965	−1.201 ***	0.179	−0.928 *	1.38 *	0.273	−1.106
	1966–1978	−0.218	0.168	−0.353	0.386	−0.135	−0.521
Geographical characteristics	Hukou type (urban Hukou)						
	Rural Hukou	1.522 ***	−0.085	0.542 ***	−1.606 ***	−0.98 **	0.626 ***
Economic characteristics	Self-evaluation of family economic status (high)						
	Low	−0.484	−0.672 *	−0.357	−0.188	0.126	0.315
	Medium	−0.144	−0.204	−0.328	−0.061	−0.184	−0.124
Educational characteristics	Educational level (Tertiary education)						
	Uneducated	0.672 *	0.655	0.33	−0.018	−0.343	−0.325
	Primary school	0.238	0.483	−0.241	0.245	−0.479 **	−0.724
	Middle school	0.232	0.213	0	−0.018	−0.232	−0.213 *
	High school	0.094	0.501	0.008	0.407	−0.086	−0.494
Social interaction characteristics	Contact with relatives (not close)						
	close	0.48	0.489 *	0.605	0.009	0.124	0.115
Sample size		2072					
Cox and Snell		0.154					
Nagelkerke		0.17					
McFadden		0.07					

Note: (1) * *p* < 0.5; ** *p* < 0.1;*** *p* < 0.001. (2) The contents in brackets represent reference groups.

## Data Availability

Publicly available datasets were analyzed in this study. This data can be found here: http://www.cnsda.org/index.php?r=projects/view&id=94525591.
